# Glioblastoma Exhibits Inter-Individual Heterogeneity of TSPO and LAT1 Expression in Neoplastic and Parenchymal Cells

**DOI:** 10.3390/ijms21020612

**Published:** 2020-01-17

**Authors:** Linzhi Cai, Sabrina V. Kirchleitner, Dongxu Zhao, Min Li, Jörg-Christian Tonn, Rainer Glass, Roland E. Kälin

**Affiliations:** 1 Neurosurgical Research, Department of Neurosurgery, University Hospital, LMU Munich, 81377 Munich, GermanySabrina.Kirchleitner@med.uni-muenchen.de (S.V.K.);; 2 Department of Neurosurgery, University Hospital, LMU Munich, 81377 Munich, Germany; 3 German Cancer Consortium (DKTK), Partner Site Munich and German Cancer Research Center (DKFZ), 69120 Heidelberg, Germany; 4 Walter Brendel Center of Experimental Medicine, Faculty of Medicine, LMU Munich, 81377 Munich, Germany

**Keywords:** TSPO, PBR, glioblastoma, LAT1, *SLC7A5*, Iba1

## Abstract

Molecular imaging is essential for diagnosis and treatment planning for glioblastoma patients. Positron emission tomography (PET) with tracers for the detection of the solute carrier family 7 member 5 (*SLC7A5*; also known as the amino acid transporter light chain L system, LAT1) and for the mitochondrial translocator protein (TSPO) is successfully used to provide additional information on tumor volume and prognosis. The current approaches for TSPO-PET and the visualization of tracer ([18F] Fluoroethyltyrosine, FET) uptake by LAT1 (FET-PET) do not yet exploit the full diagnostic potential of these molecular imaging techniques. Therefore, we investigated the expression of TSPO and LAT1 in patient glioblastoma (GBM) samples, as well as in various GBM mouse models representing patient GBMs of different genetic subtypes. By immunohistochemistry, we found that TSPO and LAT1 are upregulated in human GBM samples compared to normal brain tissue. Next, we orthotopically implanted patient-derived GBM cells, as well as genetically engineered murine GBM cells, representing different genetic subtypes of the disease. To determine TSPO and LAT1 expression, we performed immunofluorescence staining. We found that both TSPO and LAT1 expression was increased in tumor regions of the implanted human or murine GBM cells when compared to the neighboring mouse brain tissue. While LAT1 was largely restricted to tumor cells, we found that TSPO was also expressed by microglia, tumor-associated macrophages, endothelial cells, and pericytes. The Cancer Genome Atlas (TCGA)-data analysis corroborates the upregulation of TSPO in a bigger cohort of GBM patient samples compared to tumor-free brain tissue. In addition, *AIF1* (the gene encoding for the myeloid cell marker Iba1) was also upregulated in GBM compared to the control. Interestingly, TSPO, as well as *AIF1*, showed significantly different expression levels depending on the GBM genetic subtype, with the highest expression being exhibited in the mesenchymal subtype. High *TSPO* and *AIF1* expression also correlated with a significant decrease in patient survival compared to low expression. In line with this finding, the expression levels for *TSPO* and *AIF1* were also significantly higher in (isocitrate-dehydrogenase wild-type) IDH^WT^ compared to IDH mutant (IDH^MUT^) GBM. LAT1 expression, on the other hand, was not different among the individual GBM subtypes. Therefore, we could conclude that FET- and TSPO-PET confer different information on pathological features based on different genetic GBM subtypes and may thus help in planning individualized strategies for brain tumor therapy in the future. A combination of TSPO-PET and FET-PET could be a promising way to visualize tumor-associated myeloid cells and select patients for treatment strategies targeting the myeloid compartment.

## 1. Introduction

Glioblastoma (GBM) represents the majority of primary central nervous system (CNS) neoplasms in adults [1]. The prognosis of GBM is poor and has not been significantly improved in decades due to its high malignancy, immunosuppressive tumor microenvironment, and heterogeneity. Prognostic factors, such as the IDH^MUT^ and MGMT-promotor-methylation status, have been described in the past and allow for individualized treatment [2,3,4,5]. In order to address the inter-individual heterogeneity, molecular criteria were developed to stratify GBM patients into distinct genetic-subgroups (proneural, classical, and mesenchymal) [3,6]. The evolutionary dynamics of GBM, however, can generate intratumoral heterogeneity [7,8]. It is not feasible to characterize this on a genetic level, as it would require repeated extensive biopsies. Therefore, there is an urgent need for a noninvasive way to classify GBM. It was previously established that O-(2-[^18^F] fluoroethyl)-L-tyrosine-positron emission tomography (FET-PET) provides information on pathology in GBM patients and improves the visualization of GBM cells [9,10,11]. FET uptake is mediated by the amino acid transporter light chain L system (LAT1 and LAT2) [9,10]. The amino acid transporter LAT1 is overexpressed by tumor cells [12]. The exact mechanism of incorporation of 18F-FET into tumor cells is not fully understood. FET becomes internalized by the amino acid transporter, but not incorporated into proteins, and is therefore a pure amino acid transport tracer [13,14] with the ability to provide valuable information about the tumor cell [11]. However, in addition to tumor cells, the tumor microenvironment is composed of immune cells, astrocytes, oligodendrocytes, neurons, endothelial cells, pericytes, and tumor-associated myeloid cells (TAM), which support tumorigenesis and tumor expansion [15,16]. Among them, TAM constitutes up to 30% of the tumor mass [16,17]. Other than FET-PET, mitochondrial translocator protein (TSPO)-PET can visualize not only a tumor mass, but also microglial activation in TAM and potentially additional inflammatory components of GBM [18]. Translocator Protein 18kDa (TSPO) is highly expressed in myeloid cells and tumor cells, while a normal brain shows only a moderate expression. FET-PET has a good specificity for imaging tumors [12]. Therefore, the combination of both methods could provide noninvasive information about the composition of the tumor stroma and infiltrating immune cells. The tracer uptake in GBM patients for TSPO- and FET-PET has been reported to only partially overlap [19]. Some tumor areas are exclusively positive in one of the modalities [19]. Little is known about the underlying expression of TSPO and LAT1 in GBM tumor tissue and associated inflammation in the surrounding area. This study explores TSPO and LAT1 expression in human and murine GBM tissue from different GBM subtypes (Table A1). GBM stem-like cells (GSCs) were derived from human GBM of different genetic subtypes, namely proneural [20,21] and classical [22], and were cultured as spheroids until xenografting them into immunodeficient mouse brains to produce GBM. Mouse transgenic GSCs used to model genetic GBM subtypes were derived from transgenic neural precursor cells that were additionally transduced to carry typical driver mutations for proneural (*p53^KO^PDGFB*) or classical (*cdkn2a^KO^EGFRvIII*) GBM subtypes [23,24] and were kept under stem cell-like conditions until orthotopic implantation into immunocompetent mice. By the immunostaining of GBM patient samples, patient-derived xenografts, and murine genetic subtype models, we show that TSPO expression is detected in tumor cells, as well as the tumor microenvironment, especially tumor-associated myeloid cells, pericytes, and endothelial cells, while LAT1 seems to be restricted to the tumor cells themselves. We found that the TSPO level differs significantly in the various subtypes, while the LAT1 levels remain the same. In addition, we also investigated the RNA expression level for *AIF1*, the gene encoding for the myeloid cell marker protein Iba1. This analysis showed that high TSPO levels correlate with high *AIF1* levels and increased patient survival. Together, we established mouse GBM models that will allow us to study combined FET/TSPO-PET imaging to increase our understanding of the biological meaning of the patient images obtained and to unleash its prognostic potential.

## 2. Results

### 2.1. TSPO Expression is Upregulated in GBM

**Figure 1:**
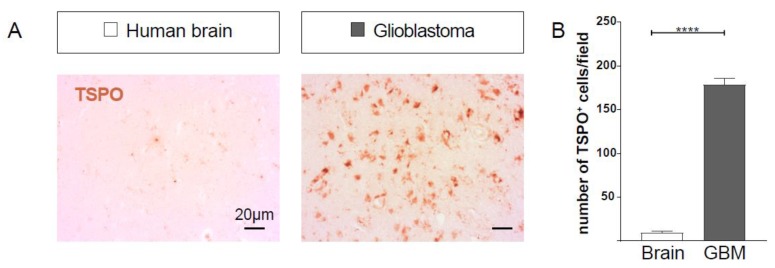
Mitochondrial translocator protein (TSPO) expression is increased in human glioblastoma compared to tumor-free brain tissue. (**A**) Immunohistochemistry of epilepsy (*n* = 4) and glioblastoma (GBM) patient samples (*n* = 4) was performed. Scale size is indicated in individual micrographs. (**B**) Number of TSPO-positive cells per field of view under a 20× objective was counted in all immunostained samples. In human GBM, significantly more cells were TSPO-positive compared to a tumor-free brain. Statistical significance (*t*-test) is indicated. **** *p* < 0.0001.

To investigate the specificity of TSPO for tumor tissue, we first analyzed patient GBM samples and compared them to those from a normal human brain. We found that in tumor-free brain tissue from epilepsy patients, few cells expressed TSPO (Figure 1A). However, the number of TSPO-positive cells largely increased in GBM tumor tissue (as seen in Figure 1A and quantified in Figure 1B).

To investigate the expression in different GBM subtypes, we next used a panel of GBM cultures from human biopsies, which were maintained under stem-like conditions and thus preserved features of GBM stem-like cells (GSCs). The respective GBM subtype of the paternal GBM biopsies was previously determined (Table A1) [20,21,22].

**Figure 2:**
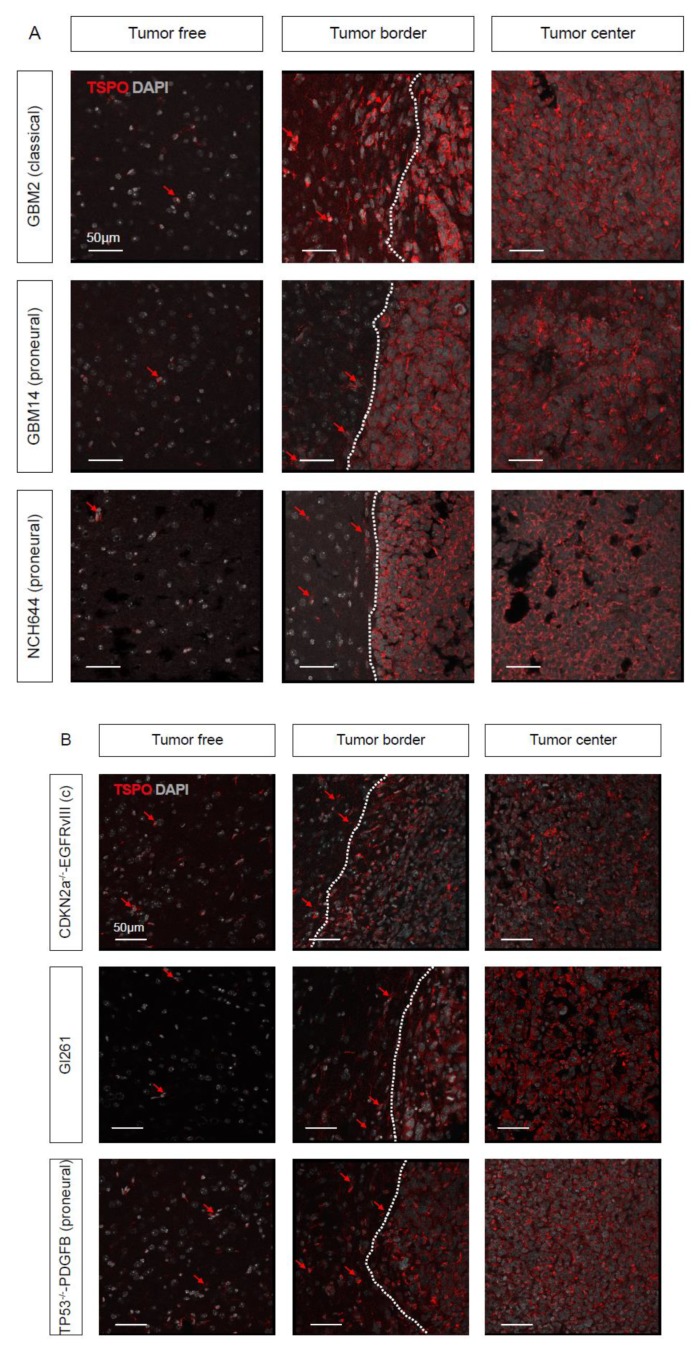
TSPO is expressed in mouse models of different GBM subtypes; (**A**) Human GBM stem-like cells (GSCs) were orthotopically xenografted in immunodeficient mice. Immunofluorescent staining against TSPO (red) and nuclear counterstaining for DAPI (gray) shows a high level of expression in classical (GBM2) and proneural (GBM14 and NCH644) human GBM subtypes. (**B**) TSPO immunofluorescent staining (red) and nuclear counterstaining (gray) of murine Gl261 and the classical (*cdkn2a^KO^EGFRvIII*) and proneural (*p53^KO^PDGFB*) murine GBMs. (**A**,**B**) Scale size is indicated in individual micrographs. A representative image from a cohort of five mice per experimental group is shown.

The implantation of these human GSCs produced patient-derived xenografts (PDX) with a high expression of TSPO in the tumor area (Figure 2A) for the classical human GBM (GBM2), as well as for the proneural human GBM (GBM14 and NCH644). In the tumor, border dispersed cells strongly positive for TSPO were visible (Figure 2A, arrows in the tumor border), likely showing invading cells and macrophages, while in the tumor-free regions, these expression patterns were weaker (Figure 2A, arrows in tumor-free areas). Next, we used a panel of genetically engineered mouse GBM cells that faithfully recapitulate the key pathological features for each GBM subtype (Table A1) [24]. By immunofluorescent staining against TSPO, the distinction between tumor-free brain and tumor samples became obvious when using murine Gl261, as well as the classical *cdkn2a^KO^EGFRvIII* or proneural *53^KO^PDGFB* GSCs (Figure 2B). In addition, in the murine GBM models, evenly dispersed TSPO-positive cells could be observed in tumor border and tumor-free areas (Figure 2B, arrows).

### 2.2. TSPO is Expressed in Parenchymal Cells of GBM

**Figure 3:**
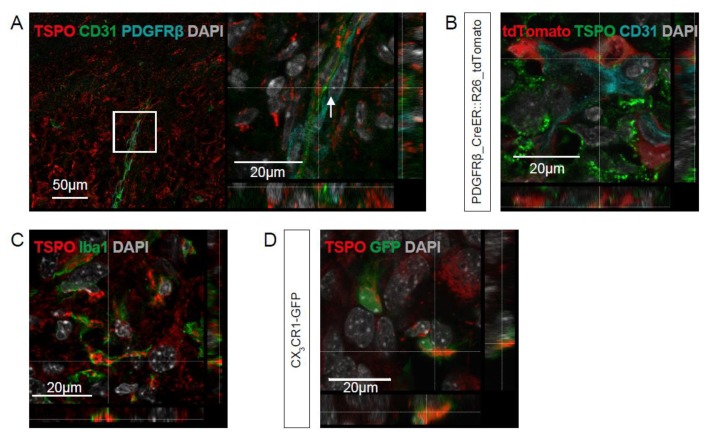
TSPO is expressed in tumor endothelia, pericytes, and myeloid cells. (**A**) Gl261 tumors grown for 3 weeks were co-stained by immunofluorescence for-anti-TSPO- (red), anti-CD31- (green), and anti-PDGFRB-antibodies (cyan) showing TSPO expression (red) in endothelia (green) and pericytes (cyan). (**B**) From Gl261 implants of PDGFRB::CreERT2,R26-tdTomato (red), mice immunofluorescent co-staining was performed using anti-CD31 (cyan) and TSPO (green), demonstrating the co-expression of TSPO in tdTomato-expressing pericytes (red). (**C**) Fluorescence staining with anti-Iba1 (green) and anti-TSPO (red) antibodies shows co-expression in tumor-associated myeloid cells. (**D**) TSPO immunofluorescence in the GL261 implant in CX3CR1-GFP mice confirms the co-expression of TSPO (red) in GFP (green)-positive microglia. (**A**–**D**) DAPI nuclear counterstaining is shown in gray; scale-size is indicated in the individual micrographs.

**Figure 4:**
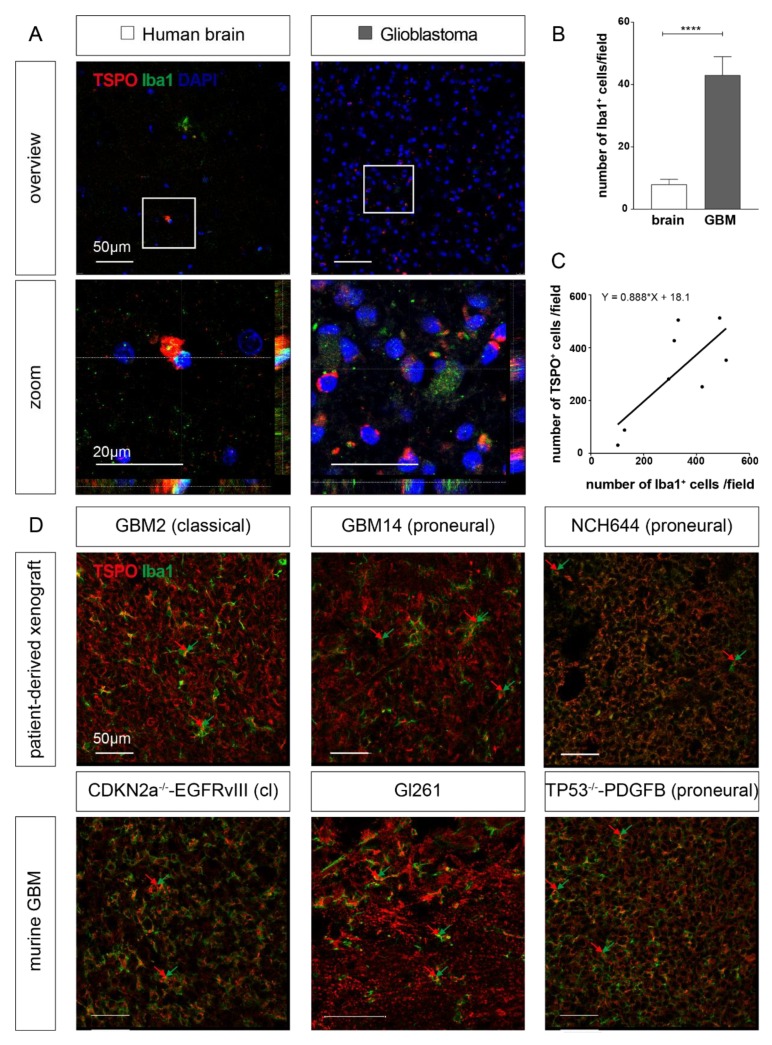
Microglia express TSPO, independent of the genetic GBM subtype. (**A**) Immunofluorescence staining of epilepsy and GBM patients. Co-staining for Iba1 (green) and TSPO (red) indicated the co-expression of both proteins in cells (zoom). (**B**) The number of Iba1-positive cells per field of view at 20x was counted. In the tumor area, significantly more Iba1-positive microglia could be found. The p-value of Student’s t test is indicated as **** *p* < 0.0001. (**C**) Immunohistochemical analysis of human GBM samples was performed and the number of positive cells was determined. A strong positive correlation between the amount of Iba1-positive cells and TSPO-positive cells was observed (*n* = 8). (**D**) Immunofluorescent staining of patient-derived xenografts (PDX) and orthotopic murine GBM implants was performed using anti-Iba1 (green) and anti-TSPO antibodies (red). In all models, TSPO-positive (red) and myeloid (green) cells were detected (arrows). (**A**,**D**) Scale size is indicated in individual micrographs.

To further investigate the identity of the cells expressing TSPO in the tumor microenvironment, we performed immunofluorescent co-staining against CD31 and PDGFRB. Interestingly, we found TSPO expression in CD31-positive endothelial cells, as well as in PDGFRB-positive pericytes attached to the newly forming vasculature within the tumor region (Figure 3A). Using the transgenic pericyte-reporter strain PDGFRB::CreERT2,R26-tdTomato [25,26], we confirmed the TSPO positivity of tdTomato-expressing pericytes (Figure 3B). Next, we also tested whether TSPO is expressed in Iba1-positive microglia and macrophages in our GBM mouse models (Figure 3C). Indeed, we found, by immunofluorescent staining, that TSPO was expressed in tumor-associated myeloid cells marked by Iba1 expression. Again, we confirmed this finding by using transgenic mice expressing a green fluorescent protein (GFP) reporter under the fractalkine receptor (CX3CR1) promotor [27,28]. Moreover, in the CX3CR1-GFP mouse, we found that microglia within the tumor expressed TSPO (Figure 3D). Next, we tested whether TSPO can also be detected in myeloid cells of patient GBM samples, as well as in the different murine and human GBM models of the various genetic subtypes. In patient GBM samples, the numbers of Iba1-positive cells were significantly higher in the tumor area compared to the tumor-free brain, as quantified on the immunohistological micrographs (Figure 4A,B). In addition, we performed an immunohistochemical analysis of GBM patient samples and counted the cell numbers for TSPO- and Iba1-expressing cells in consecutive sections. Concordantly, Figure 4C shows a strong correlation of Iba1-positive and TSPO-positive cells among human GBM. Co-staining with Iba1- and TSPO-antibodies showed that TSPO was also expressed in Iba1-positive microglia/macrophages in the tumor (Figure 4A). In the patient-derived xenografts (PDX) and murine GBMs of different genetic subtypes, a high expression of TSPO in Iba1-positive tumor-associated cells could be found (Figure 4D, arrows).

In summary, we found that TSPO expression is increased in patient GBM samples compared to normal brain tissue. The expression of TSPO can be detected in tumor cells of different experimental mouse models, as well as in tumor parenchymal cells, such as endothelial cells and pericytes of the tumor neo-vasculature, and tumor-associated myeloid cells, independent of the GBM genetic subtype.

### 2.3. LAT1 Expression is Upregulated in GBM

**Figure 5:**
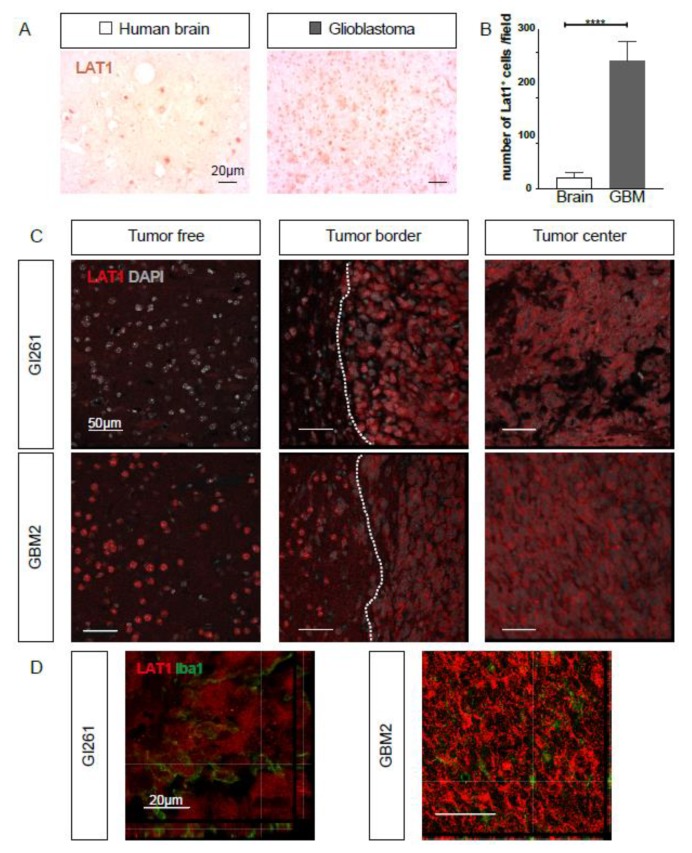
Amino acid transporter light chain L system (LAT1) expression is increased in human and murine GBM. (**A**) Immunohistochemistry of epilepsy (*n* = 4) and GBM patient samples (*n* = 4) was performed. LAT1 expression is more prominent in cells in the GBM sample compared to the control brain. (**B**) Number of LAT1-positive cells per field of view under a 20× objective was counted in all immunostained samples. In human GBM, significantly more cells were LAT1-positive compared to the tumor-free brain. Statistical significance (*t*-test) is indicated. **** *p* < 0.0001. (**C**) Immunofluorescent staining was performed against LAT1 in orthotop murine GBM (Gl261) or patient-derived xenografts (GBM2). LAT1 (red) expression was strong in the tumor boarder, while a weaker expression was visible outside the tumor area. (**D**) Immunofluorescent co-staining of orthotop murine glioblastoma (Gl261) or human xenograft (GBM2) for LAT1 (red) and Iba1 (green) shows no co-expression of LAT1 in myeloid cells. Scale size is indicated in individual micrographs.

To be able to correlate the expression pattern of TSPO with the most commonly used target for radiolabeled FET tracer for GBM, we next performed an immunohistological analysis for LAT1 (*SLC7A5*). We found that LAT1 is expressed in tumor-free human brain tissue (Figure 5A); however, the number of LAT1-positive cells is highly increased in human GBM tissue (Figure 5A quantified in Figure 5B). In orthotopically implanted murine Gl261 tumors and human GBM2 xenografts, a high expression of LAT1 could also be detected in the tumor cells, while only moderate staining outside the tumor area was visible (Figure 5C). Immunofluorescent co-staining of Iba1- and LAT1-protein in murine and human GBMs did not indicate the co-expression of LAT1 in Iba1-positive myeloid cells within the tumor area (Figure 5D).

### 2.4. TSPO and LAT1 Expression do not Correlate in a Big Cohort of GBM Patient Samples

**Figure 6:**
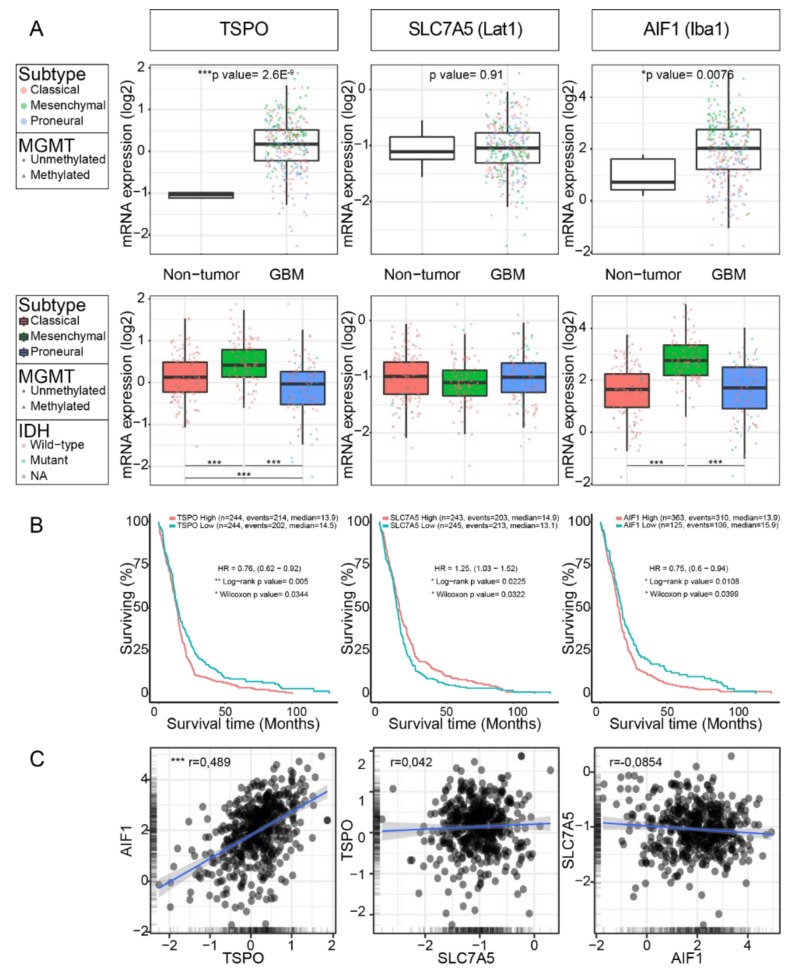

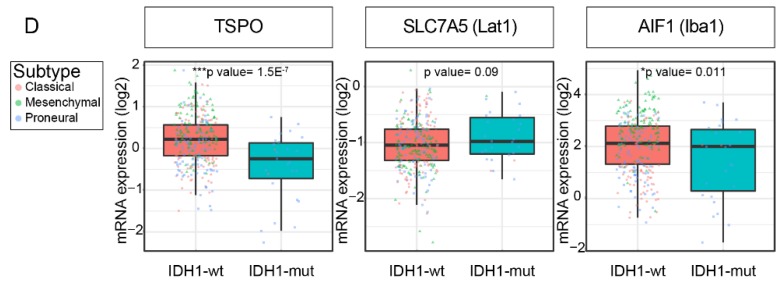
*TSPO*, solute carrier family 7 member 5 (*SLC7A5*), and *AIF1* expression analysis in a TCGA GBM cohort. Patient GBM samples of the TCGA database were analyzed using GlioVis [29]. (**A**) Gen expression levels in GBM compared to the tumor-free brain showed that expression levels for *TSPO* and *AIF1* were significantly higher in GBM. *P*-value of Student’s t test is indicated (upper panel). Significant differences between the gene expression of *TSPO* and *AIF1* were also detected for GBM subtypes. Statistical significance (Tukey’s Honest Significant Difference) is indicated, *** *p* < 0.0005 (lower panel). (**B**) Survival of patients with a high or low expression of *TSPO*, *SLC7A5*, or *AIF1* was analyzed. High *TSPO* or *AIF1* expression was associated with significantly shorter survival, while high *SLC7A5* expression levels were favorable for survival. Patient number indicated in graph. (**C**) A correlation between the genes *TSPO*, *SLC7A5*, and *AIF1* was performed. A significant positive correlation between *TSPO* and *AIF1* (Pearson’s *r* = 0.489) was observed, while no correlation between the other pairs could be seen. (**D**) Expression levels for *TSPO* and *AIF1* were significantly higher in IDH^WT^ GBM than in IDH^MUT^. P-value of Student’s *t*-test is indicated.

The TCGA glioblastoma dataset was analysed for the expression of *TSPO* and *SLC7A5* (=LAT1), encoding for the molecular targets of TSPO- and FET-PET, and for *AIF1*, as we found a high TSPO level in microglia and macrophages. By conducting this analysis, we found a significant increase in the gene expression of *TSPO* and *AIF1* in GBM compared to that of healthy brain tissue (Figure 6A). Within the GBM subtypes (as defined by Wang et al. [6]), significant differences in the expression of *TSPO* and *AIF1* genes could be found (Figure 6A). Interestingly, Lat1 expression over the many GBMs in the TCGA dataset was not significantly changed compared to that of the non-tumor area (Figure 6A) and no variation in expression could be detected in the different genetic subtypes. Looking at patient survival, we found that high TSPO or *AIF1* expression was associated with significantly shorter survival, while high *SLC7A5* (=LAT1) levels were correlated with an increased survival (Figure 6B). Based on this finding, we analysed an additional GBM cohort consisting of 12 patient samples by immunohistochemistry for TSPO- or Iba1-positive cells and quantified numbers (Supplementary Figure S1A,B). In line with the TCGA data, we found that patients with a shorter survival (less than 2 years) showed a higher number of TSPO- or Iba1-positive cells. Next, we also performed a correlation analysis of the expression levels of the three genes *TSPO*, *SLC7A5*, and *AIF1* in the TCGA GBM dataset and identified a significant positive correlation between *TSPO* and *AIF1* (Figure 6C). When performing the same analysis by different genetic subtypes, this positive correlation was also significant (data not shown). Finally, we also investigated the levels of *TSPO* and *AIF1* expression in GBMs in the TCGA dataset that were IDH ^WT^ or IDH^MUT^ (Figure 6D). Interestingly, we found that the IDH^WT^ GBMs also showed significantly higher expression levels in *TSPO* and *AIF1*.

## 3. Discussion

In this study, we performed an expression analysis of TSPO and LAT1—the target structures of molecular imaging modalities—in patient GBM samples, as well as in orthotopic GBM mouse models, from patient-derived and murine transgenic GBM cells, modeling the different GBM genetic subtypes. TSPO has been shown to be a robust and sensitive biomarker for microglial activation in neurodegenerative conditions [30] and increased TSPO levels in PET were found in the progression of mild cognitive impairment and Alzheimer’s disease [31,32]. In human addiction studies of methamphetamine use, an increased TSPO-PET signal was found and correlated with TSPO upregulation in reactive glial cells and activated microglia [33]. Additionally, in almost all types of CNS pathologies, TSPO levels are increased, and in some psychiatric disorders, such as schizophrenia, the TSPO signal in PET is decreased [34]. Although TSPO ligands are widely used in molecular imaging in all sorts of CNS pathologies, little is known about the role of TSPO expression and neuroinflammation in glioblastoma patients and PET imaging with TSPO radioligands is not yet standard for the imaging of brain tumor patients [18,19,35,36,37]. In this study, we could show that the expression of TSPO is higher in brain tumor samples compared to those from epilepsy patients. In patient-derived xenograft and transgenic murine GBM models, TSPO is strongly upregulated in the tumor area compared to normal brain regions. The highest TSPO intensity was observed in the tumor cells themselves in several human and murine GBM models. Interestingly, TSPO was found to be expressed in Nestin-positive neural stem/progenitor cells in the subventricular zone and at the base of the rostral migratory stream in mice [38]. TSPO expression also co-localizes at low levels with glial fibrillary acidic protein (GFAP) in neural stem/progenitor cells in the subventricular zone, confirming the presence of TSPO in neural stem/progenitor cells [38]. As neural stem/progenitor cells are the putative source of GBM cells, this may be one reason why our patient-derived GBM cells and murine transgenic GBM cells (derived from NPCs) uniformly express TSPO. TSPO expression per se may not be a discriminating criterion, but high TSPO expression may be informative.

However, not only tumor cells express TSPO; microglia/macrophages, endothelial cells, and pericytes were also strongly positive for TSPO in the tumor regions of our GBM models. This finding is in line with previous studies that detected the expression of TSPO in vascular structures of a normal brain [38]. TSPO ligands bind in larger blood vessel walls [39] and the co-localization of TSPO with PDGFRB-positive pericytes and smooth muscle cells was observed [38,40]. The association with CD31-positive endothelial cells was also previously observed in vessels of different sizes down to capillaries [38]. TSPO-positive vasculature was present in brains, regardless of disease, suggesting that endothelial TSPO might be constitutive [40]. This observation is important because it relates to the binding and distribution of TSPO ligands in the brain parenchyma [40]. In our study, the highest TSPO levels were seen in the parenchymal cells in the tumor regions, indicating an upregulation of TSPO in the tumor neo-vasculature and tumor-associated myeloid cells.

Besides the representation of GBM-patient heterogeneity by genetic subtypes, our novel murine GBM models provide the advantage that immunocompetent mice can be inoculated, in contrast to T-cell deficient mice used for patient-derived GBM cells (24). Although GBM are notoriously low in immunogenicity, this difference could impact TSPO expression levels and is worth studying in the future. For example, our models can be employed to investigate the effect that an immune therapy treatment might have on TSPO-PET imaging.

Studies have also described that TSPO was not detectable in oligodendrocytes stained by anti-MBP-antibody or CD11b-positive microglia of a healthy brain [38,41]. Increased TSPO expression, however, was detected in a pro-inflammatory brain environment [42]. For example, in acute multiple sclerosis (MS), the majority of TSPO-positive cells were macrophages and microglia. Additionally, in Alzheimer’s Disease (AD), most TSPO-positive cells were also Iba1-positive [40]. In HIV encephalitis, many TSPO-positive cells were co-stained with CD68, a macrophage-lineage marker [40]. High TSPO expression in astrocytoma has been shown to correlate with shorter survival and higher proliferation [43]. In astrocytoma, TSPO is expressed in Iba1+ cells, but not GFAP+ astrocytes [44], while reactive astrocytes can contribute to the signal, in addition to reactive microglia [41]. In chronic silent MS lesions, reactive astrocytes can also express TSPO [40]. In this study, we show (after the quantification of immunohistochemical data) a strong correlation between the expression of TSPO and Iba1 in a small patient cohort. These findings are in line with our conclusion from the analysis of the TCGA GBM dataset. Most interestingly, high expressions of both molecules emerged as unfavourable prognostic markers. As IDH^WT^ GBM tumors have a less favourable prognosis than IDH^MUT^ GBM, we interrogated the TCGA GBM dataset for the expression levels of our markers in IDH^WT^ against IDH^MUT^ GBM. *TSPO* and *AIF1* expression levels were significantly increased in IDH^WT^ compared to IDH^MUT^ GBM.

Immunostaining for LAT1 produced a less intense and more punctate staining pattern compared to immunofluorescence for TSPO. The expression of LAT1 protein was higher in GBM than in healthy brain tissue, although gene expression in TCGA data was not altered. The highest LAT1 intensity was observed in the tumor cells themselves in the various human and murine GBM models studied here. It has previously been shown that LAT1 is expressed in tumor cells and in proximity to the vascular endothelium [45,46]. LAT1 mRNA and protein expression varies in GBM [47] and the expression of LAT1 was reported to be higher in infiltrating glioma cells than in cells located in the tumor center [46]. A strong positive correlation between LAT1 and Ki67 [48] and a correlation between high LAT1 protein expression with poor survival were demonstrated [49]. This contrasts with our analysis of the TCGA dataset, which showed a favorable outcome for high LAT1 mRNA expression. Even though the prognostic value of LAT1 is not conclusive, the fact that FET concentrates in malignant cells, possibly due to asymmetrical recognition by the LAT1 transporter [12], has made it a valuable target for clinical PET imaging [11].

While LAT1 is mainly expressed in tumor cells, we and others have found that TSPO is expressed not only by tumor cells, but additionally in microglia/macrophages, endothelial cells, and pericytes of the tumor microenvironment. Therefore, the combination of FET-PET and TSPO-PET might be a promising way to visualize not only the tumor, but also the reactive tumor microenvironment, such as the tumor-infiltrating myeloid cells and the tumor neo-vasculature. Additionally, the TSPO level might show prognostic relevance, specifically in IDH^WT^ GBM. For that group of patients, our results would propose that a high expression of TSPO could be a marker for decreased survival. Further studies using the novel GBM models described here, in combination with a prospective GBM patient cohort, will allow the interpretation of molecular imaging data to be improved and different PET-signals with pathological features of individual tumors and the inter-patient and intratumoral GBM heterogeneity to be related.

## 4. Materials and Methods

### 4.1. Cell Culture

GSCs were derived from human glioblastoma biopsies, as previously described for NCH644 [20] and GBM14 [21]. Murine transgenic *cdkn2a^KO^EGFRvIII* and *p53^KO^PDGFB* GBM cells were previously generated [24]. All cells were cultured as spheroids in DMEM-F12 (Cat. 11320-074, ThermoFisher Scientific, Waltham, MA, USA) supplemented with 1× B27 (Cat. 17504-044, ThermoFisher Scientific, Waltham, MA, USA), 1% penicillin-streptomycin (Cat. 151140-122, ThermoFisher Scientific, Waltham, MA, USA), 10 ng/mL epidermal growth factor (EGF; Cat. 236-EG; Biotechne; Minneapolis, MN, USA), and 10 ng/mL fibroblast growth factor (FGF; Cat. 100-18B PeproTech, Hamburg, Germany). Murine Gl261 glioma cells were obtained from the National Cancer Institute, NCI-Frederick (Tumor Cell Repository), and maintained under adherent conditions in DMEM containing 1× MEM non-essential amino acids (Cat. 11140-035, ThermoFisher Scientific, Waltham, MA, USA), 1% penicillin-streptomycin, and 10% fetal bovine serum (Cat. 102270-106, ThermoFisher Scientific, Waltham, MA, USA). Human GBM2 GSCs were cultured as spheroids with Neurocult Basal Medium (Cat. #05700, StemCell, Grenoble, France) with 10% Neurocult Proliferation Supplement (Cat. #05701, StemCell, Grenoble, France), 1% penicillin-streptomycin, 10ng/mL hFGP, and 10ng/mL hEGF [22]. All cells were maintained at 37 °C in a humidified atmosphere of 95% O_2_ and 5% CO_2_. GSCs were validated repeatedly by short tandem repeat (STR) fingerprinting (Eurofins Medignomix Forensik, Munich, Germany) and were regularly tested for mycoplasma contamination by PCR.

### 4.2. Animal Experiments

All experiments were conducted in accordance with the National Guidelines for Animal Protection Germany, with approval of the local animal care committee of the Government of Oberbayern (projects 55.2-2532-2012/2018). Pericyte reporter mice PDGFRB::CreERT2,R26-tdTomato were created by cross breeding B6.cg_Tg(pdgfb-cre/ERT2)6096Rha/J mice [50] expressing a fusion protein of cre-recombinase and a modified estrogen-receptor (cre-ER2) under the PDGFRB promotor with a cre-reporter line (B6.Cg-Gt(ROSA)26Sortm9(CAG-tdTomato)Hze/J) [26]. Mice containing a knock-in of green fluorescent protein (GFP) in the gene (*CX3CR1*) encoding for the fractalkine receptor (B6.129P2(Cg)-Cx3cr1tm1Litt/J) [27] were used to label the myeloid cells in the brain. All mice were purchased from the Jackson Laboratory, bred on C57Bl/6J background, and genotyping was performed as previously described. Immunodeficient Foxn1nu/nu or B6.129S6-Rag2tm1Fwa mice were used for the xenografting of human GBM cells, as described previously [24]. Animals were kept in suitable cages with ad libitum access to water and food in a 12-h light/dark cycle at the standardized animal house of the Walter Brendel Centre for Experimental Medicine, LMU Munich. Mice were sacrificed at defined presymptomatic time points.

### 4.3. Tumor Inoculation

Mice received i.p. 7 µL/g body weight of a mixture of 0.1% xylazine (Rompun 2%; Bayer, Leverkusen, Germany) and 1.5% ketamine (Ketavet; Zoetis, Berlin, Germany) in 0.9% NaCl. Their corneas were covered with a moisturizing cream (Bepanthen; Bayer). After disinfection with 10% potassium iodide solution, a midline incision was made in the skin of the skull with a scalpel. Mice were immobilized on a stereotactic frame (David Kopf Instruments, Tujunga, CA, USA). After drilling a hole into the skull with a 23G needle tip at the puncture point 1.5 mm anterior and 1.5 mm right of the bregma, 1 μL PBS or GBM cells (1 × 10^5^ Gl261 or human GBM cells/µL and 5 × 10^4^ murine GBM cells/µL) was slowly injected into the mouse brain at a 3 mm depth within 2 min using a 22G Hamilton syringe (Hamilton, Bonaduz, Switzerland). Afterwards, the syringe was removed in 1 mm steps per minute and the skin was sutured. Tumors were grown for 3 weeks for murine cells and for up to 6 weeks for human GBM cells, as previously reported [24].

### 4.4. Mouse Brain Tissue Preparation

Mice were transcardially perfused with 1 x PBS followed be 4% PFA solution under anesthesia. The mouse brain was collected and fixed in 4% for 24 h at 4 °C and was then dehydrated in 30% sucrose for ≥24 h at 4 °C. Next, the brain was embedded in Cryomatrix^®^ (Cat. 6769006; ThermoFisher Scientific) and gently frozen in isopentane to −80 °C. Tissue samples were prepared as horizontal sections (40-μm-thick) using a horizontal sliding microtome. Floating sections were stored at −20 °C in cryoprotectant (ethylene glycol, glycerol, and 0.1 M PO4 buffer in a 1:1:2 solution at pH 7.4) protected from light.

### 4.5. Fluorescent Immunohistochemistry and Confocal Microscopy

Floating sections were washed three times for 5 min with PBST (0.1% Tween-20 in PBS), and the endogenous peroxidase was blocked with Dako endogenous enzyme block (Dako S2003) for 10 min at room temperature, washed with PBST three times, and blocked for 1 h at room temperature in 5% normal donkey serum (NDS; Cat. 017-000-121; Jackson Immuno-Research, Westgrove, PA, USA) and 0.3% Triton-X (Cat. 93418; Fluka) in PBS or Protein Blocking Reagent (Cat. X0909, Dako, Agilent Technologies, Santa Clara, CA, USA). The sections were then incubated overnight at 4 °C with the primary antibodies (Table A2) diluted in Dako antibody diluent (Cat. S3002, Dako, Agilent Technologies, Santa Clara, CA, USA). The next day, after washing the sections, they were incubated for 2 h at room temperature with the secondary antibodies (Table A2) diluted in Dako antibody diluent. After washing sections, they were incubated with fluorophore coupled streptavidin (Table A2) for 2 h at room temperature. Nuclei were stained with 2 µg/mL DAPI (Cat. 32670; Fluka). Finally, sections were mounted on glass slides (SuperfrostTM Plus, R. Langenbrinck GmbH, Emmendingen, Germany) and air dried for 10 min. Tissue was mounted in Fluorescent Mounting Medium (Cat. S3023; Dako) and covered (high-precision microscope cover glasses No1.5H, Schott, Germany). Confocal microscopy was performed at the bioimaging core facility of the Biomedical Center (LMU Munich) with a Leica SP8X WLL microscope, equipped with a 405 nm laser, WLL2 laser (470–670 nm), and acusto-optical beam splitter. Images were acquired with a 63 × 1.4 objective for the visualization of single cells and subcellular structures, and the image pixel size was 80 nm. Conversely, they were acquired with a 40 × 1.3 objective for the visualization of changes at the tumor border, and the image pixel size was 80 nm. The following fluorescence settings were used: DAPI (excitation 405; emission 410–470), GFP (489; 492–550), Cy3 (558; 560–600), and Cy5 (650; 652–700). Recording was sequentially conducted to avoid bleed-through. For each experiment, imaging was administrated with appropriate settings based on the standardized negative and positive groups. Images were processed with Leica Application Suite X (LAS X) Version 3.4.1 (Leica Microsystems, Wetzlar, Germany) and Fiji [51].

### 4.6. Immunohistochemistry of Human Specimens

HOPE-embedded human tissue was obtained from the Neurosurgery Department of the University Hospital, LMU Munich (project 18-304), cut into 15 µm thick slices, and mounted on slides. Deparaffinization and rehydration were performed with Roti-Histol (Carl Roth, Germany) and graded alcohol, fixation in acetone at −20 °C for 10 min, and cooking at 100 °C in Citrate Buffer (1.8 mM Citric acid, and 8.2 mM tri-Natriumcitrate-Dihydrate, adjusted to PH 6.0 with 2 mM NaOH) for 20 min. Endogenous peroxidase was blocked with the Dako endogenous enzyme block (Dako S2003) for 10 min at room temperature. Incubation with the primary antibody was performed as described above. The next day, after three washes in PBS to remove unbound antibodies, the sections were incubated with secondary biotin donkey anti-rabbit, anti-rat, or anti-mouse antibodies (Table A2) for 3 h and then for 1 h at room temperature with streptavidin-conjugated horseradish peroxidase (1:200, Cat. SA25 5004, Vector Laboratories). After being washed in PBS, the sections were stained with DAB substrate, according to the manufacturer’s instructions (Cat. DC137C100DCS, Innovative Diagnostik-Systeme, Hamburg, Germany). The slides were then rinsed with tap water, dehydrated with a graded series of ethanol (70%, 80%, 96%, and 100%), cleared two times with xylene, and covered with Roti^®^ Histokit II mounting medium (Cat. 6640.1, Carl Roth GmbH, Karlsruhe, Germany). A Breukoven BMS D1-223A light microscope was used for inspecting and taking images after IHC staining. Cell numbers were counted under a 20X objective lens with the light microscope or ImageJ/Fiji, and Java-based open source software was used for automated cell counting. This software provides efficient and customizable quantification methods for microscopy images [52].

### 4.7. GlioVis Analysis

Data from The Cancer Genome Atlas (TCGA) Project was analyzed using GlioVis (http://gliovis.bioinfo.cnio.es/), a web application for data visualization and analysis, to explore previously published brain tumor gene expression datasets [29]. We only included adult patients from the TCGA-GBM dataset whose tumors had Agilent-4502A RNA expression data for our genes of interest available. Tukey’s Honest Significant Difference (HSD) test was performed. *P*-values of the pairwise comparisons are indicated as *** *p* < 0.001; ** *p* < 0.01; * *p* < 0.05; and ns, not significant.

### 4.8. Statistical Analysis

GraphPad PRISM 5 was used for all statistical analysis of immunohistochemistry. The number of individuals, replicates, and or repetitions of independent experiments are indicated in the text of figures. When comparing two groups, values are reported as the mean ± SD and an unpaired, non-parametric Student’s t test was used to determine statistical significance. Statistical significance was assumed if *p* < 0.05; *P*-values are indicated in figures as *** *p* < 0.001; ** *p* < 0.01; * *p* < 0.05; and N.S.: no significance.

## Supplementary Materials

Supplementary materials can be found at https://www.mdpi.com/1422-0067/21/2/612/s1.

## Abbreviations

*AIF1*	Allograft inflammatory factor-1 (also known as Iba1)
CX3CR1	C-X3-C Motif Chemokine Receptor 1 (also known as fractalkine receptor)
GBM	Glioblastoma
GSC	Glioblastoma stem-like cell
IHC	Immunohistochemistry
Iba1	Ionized calcium-binding adapter molecule 1 (encoded by the gene *AIF1*)
LAS X	Leica Application Suite X
LAT1	Amino acid transporter light chain L system (encoded by the gene *SLC7A5*)
PBR	Peripheral benzodiazepine receptor (encoded by the gene TSPO)
PDGFRB	Platelet-derived growth factor receptor beta
PET	Positron emission tomography
*SLC7A5*	Solute carrier family 7 member 5 (also known as LAT1)
TAM	Tumor-associated myeloid cell
TCGA	The Cancer Genome Atlas
TSPO	Translocator protein of 18 kDa (also known as PBR)

## Appendix A

**Table A1:** Glioblastoma stem-like cell (GSC) cultures.

Glioma Cell Line and GSC Cultures^1^	Species	GBM Subtype
Gl261 glioma cell line	murine	
*cdkn2a^KO^EGFRvIII* GBM-	murine	classical
*p53^KO^PDGFB GBM*	murine	proneural
GBM#2	human	classical
GBM#14	human	proneural
NCH644	human	proneural

^1^ Generation is described in [20,21,22,24].

**Table A2:** Antibodies.

Target	Label	Species	Catalog	Company	Concentration
anti-TSPO		rabbit	ab109497	abcam	1:250
anti-LAT1		rabbit	LS-C415524	Biozol	1:100
anti-Iba1		goat	ab5076	abcam	1:400
anti-CD31		rat	cat. 550274	BD Biosciences	1:50
anti-PDGFRβ		goat	AF1042	R&D Systems	1:100
anti-rabbit	biotin	donkey	Cat. 711-065-152	Jackson Immuno-Research	1:250
anti-rabbit	AF488	donkey	Cat. A-21206	ThermoFisher Scientific	1:500
anti-goat	biotin	donkey	B7024	Sigma	1:250
anti-goat	AF488	donkey	Cat. 705-545-147	Jackson Immuno-Research	1:500
anti-goat	AF647	donkey	Cat. 705-605-003	Jackson Immuno-Research	1:500
anti-rabbit	AF594	donkey	Cat. A-21207	ThermoFisher Scientific	1:500
anti-rat	biotin	donkey	Cat. 712-065-150	Jackson Immuno-Research	1:250
streptavidin	AF488		Cat. 016-540-084	Jackson Immuno-Research	1:500
streptavidin	AF594		Cat. 016-580-084	Jackson Immuno-Research	1:500
