# Supplementary material for: Glioblastoma Exhibits Inter-Individual Heterogeneity of TSPO and LAT1 Expression in Neoplastic and Parenchymal Cells
Source: Int J Mol Sci. 2020 Jan 17;21(2):612. doi: 10.3390/ijms21020612 (PMC7013601; doi:10.3390/ijms21020612)
Supplement: Supplementary file 1 [file ijms-21-00612-s001.pdf]

International Journal of
Molecular Sciences

MDPI

1. Supplementary Materials:

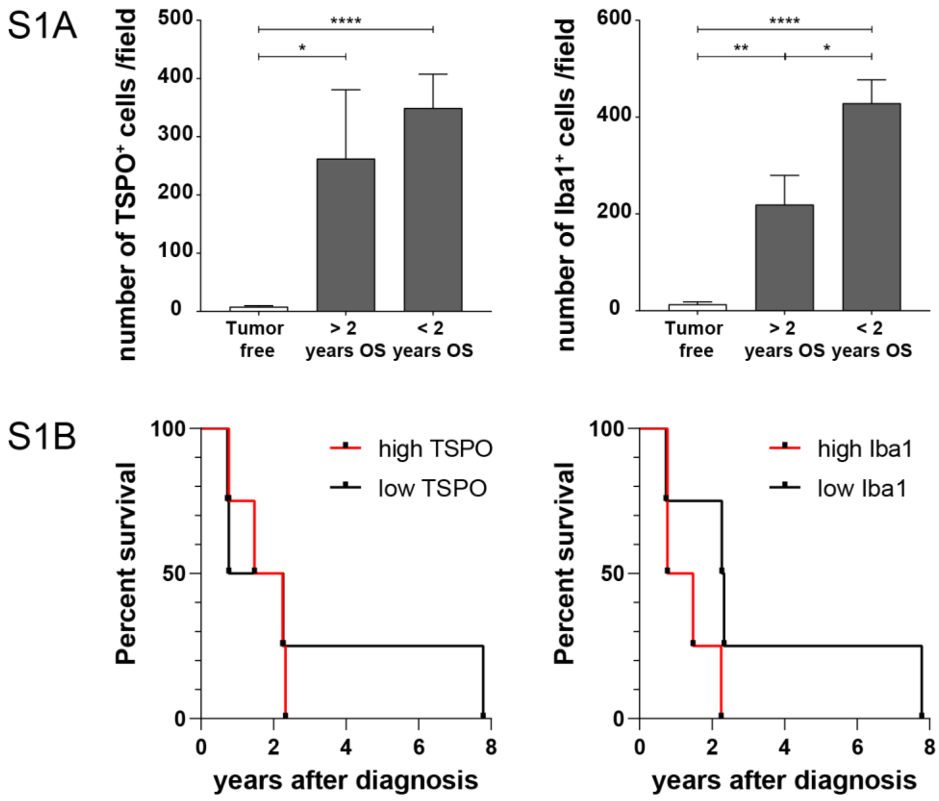

Figure S1. TSPO and Iba1 expression is increased in human glioblastoma compared to tumor free brain tissue. (**A**) Immunohistochemistry of epilepsy and GBM patient samples with short and long overall survival ($n = 4$) were performed. Number of TSPO (left) or Iba1 (right) positive cells per field of view was determined. In human GBM significantly more cells were TSPO or Iba1 positive compared to tumor free brain. Patients with longer overall survival showed significantly less Iba1 cells. Statistical significance (t-test) is indicated as as **** $p < 0.0001$; ** $p < 0.01$; * $p < 0.05$. (**B**) Overall survival of patients with high or low TSPO (left) or Iba1 (right) expression in the IHC staining showed no significant difference ($n = 4$).
